# Prognostic value of APTT combined with fibrinogen and creatinine in predicting 28-Day mortality in patients with septic shock caused by acute enteric perforation

**DOI:** 10.1186/s12893-023-02165-6

**Published:** 2023-09-12

**Authors:** Shuiqiao Fu, Wenqiao Yu, Qinghui Fu, Zhipeng Xu, Shaoyang Zhang, Ting-bo Liang

**Affiliations:** 1 The Department of SICU, The First Affiliated Hospital, Zhejiang University School of Medicine, Qingchun street 79th, Hangzhou, 310003 Zhejiang Province China; 2 The Department of Emergency, The First Affiliated Hospital, Zhejiang University School of Medicine, Qingchun street 79th, Hangzhou, 310003 Zhejiang Province China; 3 The Department of Hepatobiliary and Pancreatic Surgery, the First Affiliated Hospital, Zhejiang University School of Medicine, Qingchun street 79th, Hangzhou, 310003 Zhejiang Province China

**Keywords:** Septic shock, Acute enteric perforation, Activated partial thromboplastin time, Creatinine, Fibrinogen

## Abstract

**Background:**

Septic shock is one of the leading causes of mortality in intensive care units. This retrospective study was carried out to evaluate the association of clinical available factors with 28-day mortality.

**Patients and method:**

In this observational study, patients with perioperative septic shocks secondary to intra-abdominal infection caused by enteric perforation were included. A total of 328 sepsis patients were admitted to the surgical intensive care units from January 2012 to December 2016. A total of 138 patients met the enrolment criteria and were included in the study. The data of demographic, clinical and laboratory were all recorded.

**Result:**

All these 138 patients received abdominal surgery prior to surgical intensive care units caused by acute enteric perforation. These patients were all met the diagnostic criteria of septic shock according to Sepsis-3. Statistical analysis showed that lactic acid, blood platelet, fibrinogen, creatinine and activated partial thromboplastin time were found to be associated with 28-day mortality. A combination of serum activated partial thromboplastin time combined with fibrinogen and creatinine could predict in-hospital 28-day mortality. The area under the curve of serum activated partial thromboplastin time combined with fibrinogen and creatinine is 0.875 (0.806–0.944).

**Conclusion:**

In conclusion, this pilot study demonstrated that these factors can predict the prognosis of septic shock caused by enteric perforation. In order to reduce the mortality, surgeons and intensive care units physician may consider these data in perioperative period.

**Supplementary Information:**

The online version contains supplementary material available at 10.1186/s12893-023-02165-6.

## Introduction

Gastrointestinal perforation is one of the most common abdominal surgical emergencies requiring emergency operation in hospital. It can develop to sepsis, septic shock and multiple organ dysfunction syndrome (MODS). The morbidity and mortality of patients who develop septic shock after surgery of enteric perforation is still high.

Moreover, sepsis is one of the leading causes of mortality in intensive care units (ICUs) [1]. With its growing incidence rate and high mortality rate, sepsis is a very important sociosanitary problem [2]. Patients with sepsis often need to consume more medical resources. It is estimated that the United States has paid between $16 billion and $25 billion annually for sepsis-related medical care. Sepsis seriously threatens human health and brings a huge economic burden to society [3]. Data from previous studies showed that the incidence of severe sepsis (up to 300 cases per 100 000 adults) is increasing, although mortality has declined with the improvement of medical care [4]. Septic shock refers to hypotension in sepsis patients after full fluid resuscitation, with a need for vasoactive drugs to maintain blood pressure > 65mmHg, a level of blood lactic acid that is more than 2 mmol/L and organ dysfunction [5]. Therefore, the timely diagnosis and treatment of sepsis is very important for the prognosis of patients [6].

Septic shock caused by acute enteric perforation is a severe problem in ICU. Although blood culture is the gold standard for diagnosing sepsis, it can take more than 72 h to obtain results [7]. The prognosis to severity of the disease is also critical. There are also some clinical indexes can used to clinical prognosis of sepsis including: white blood cells, C-creative protein, procalcitonin, fever and increased neutrophil percentage [8]. But these indexes are not sensitive enough to infection even if they can be easy to detect. Meanwhile, the reports on the evaluation of prognostic indexes of septic shock caused by gastrointestinal perforation are rare. Thus, in this study, our objective was to determine a more meaningful combination of fibrinogen, creatinine and activated partial thromboplastin time for the evaluation of patients with septic shock caused by gastrointestinal perforation through the analysis of commonly used clinical parameters.

## Materials and methods

### Patients and Study Design

This was a retrospective study. This study was carried out in accordance with “WMA Declaration of Helsinki 2013 Ethical Principles for Medical Research Involving Human Subjects”. Researchers retrieved and collected patients with septic shock caused by enteric perforation in our hospital, a large academic medical center. Patients with septic shock caused by enteric perforation who were transferred to the ICU were included in the study. These patients all underwent urgent surgery. Patients who met the following criteria were excluded: 1. younger than 18 years old; 2. neoplastic disease; 3. pregnancy or severe immune system disorders; 4. end-stage chronic disease or cancer; 5. secondary operation and repeated admission to the same ICU was needed; 6. chronic renal failure and chronic liver dysfunction; 7. septic shock caused by an infection outside the abdomen; 8. criteria for septic shock (Sepsis-3) not met within 24 h after admission to the ICU. This study protocol was approved by the institutional review board of the First Affiliated Hospital Zhejiang University College of Medicine. For the included patients, the sex, age, diagnosis, laboratory test results, liquid volume, organ dysfunction, ICU time, hospital time and rate of 28-day survival were collected.

### Diagnosis and treatment

Sepsis and septic shock were defined according to the Third International Consensus Definitions for Sepsis and Septic Shock (Sepsis-3) [3]. Septic shock refers to hypotension in sepsis patients after full fluid resuscitation, with vasoactive drugs are needed to maintain blood pressure > 65mmHg and blood lactic acid level that is more than 2 mmol/L. All patients who suffered enteric perforation received a completed physical examination. Blood specimens were taken from these acute abdomen patients for chemical evaluations, including white blood cell counts (WBC), blood platelet (PLT), procalcitonin (PCT), creatinine (Cr), C reactive protein (CRP), lactic acid, prothrombin time (PT), activated partial thromboplastin time (APTT), fibrinogen, D-dimer, hematokrit (HCT), neutrophil lymphocyte ratio (NLR), platelet lymphocyte ratio (PLR) and brain natriuretic polypeptide (pro-BNP) in the first 24 h.

All the patients who were included in this study received a surgical operation and standard medical treatment according to the recent international guidelines of intensive care management: including fluid resuscitation, anti-infectious therapy with antibiotic, organ function support (ventilator support, continuous renal replacement therapy) and vasoactive drugs. The day that the patients was transferred into the ICU was considered the first day. Patients who survived more than 28 days and left the hospital healthy were considered survivors. The Sequential Organ Failure Assessment scores (SOFA) of every patient were calculated based on the worst value including oxygenation index, platelet count, bilirubin, mean arterial blood pressure, Glasgow Coma Scale, creatinine and urine volume and recorded in the first 24-hour. Acute Physiology and Chronic Health Evaluation II (APACHE II) consists of acute physiological score, age score, Glasgow Coma Scale and chronic health score. According to the previous studies, the severity of the patient was graded by SOFA score and APACHEII score as follows: 1. SOFA scores: <6, 6–10, > 10; and 2. APACHE II scores: <16, 16–24,>24 [9, 10]. The main outcome measure was 28-day mortality after ICU admission.

### Statistical analysis

Statistical analyses to identify risk factors were performed using SPSS 19.0 for Windows (SPSS, Chicago, IL). Categorical variables were grouped based on clinical findings, and decisions on the groups were made before modelling. Continuous variables were compared using the Students’t test or the Mann-Whitney U test for variables that did not conform to the normal distribution. Survival curves were depicted using the Kaplan-Meier method and compared using the log-rank test. Cox regression analysis was used for multivariate analyses. The analysis result of COX in this study was visualized based on the results of multivariate analysis and by rms package in R version 3.2.5. Receiver operating characteristic (ROC) curves and area under the curve (AUC) were used to compare the prognostic accuracy of different indicators. The most accurate cutoff value was calculated by the Youden Index. The value of *p* < 0.05 was considered statistically significant in statistical analysis.

## Results

### Patient characteristics

Between January 2012 and December 2016, 328 patients with a primary diagnosis of septic shock were admitted to the surgical intensive care unit (SICU) at our hospital. According to inclusion and exclusion criteria, 138 patients with septic shock caused by enteric perforation requiring emergency surgery were selected. There were 94 survivors and 44 non-survivors. All 44 patients died due to hemodynamic failure at last. All of these patients needed norepinephrine to maintain their blood pressure. 39 of these patients developed acute renal failure. One patient developed hepatic injury with a total bilirubin greater than 204umol/L. Nine patients developed Coagulation function dysfunction with platelets less than 20*10^9/L. 33 patients developed respiratory failure with oxygenation index less than 200. The baseline characteristics of these patients can be seen in Table 1.

**Table 1:** Baseline characteristics

Variable	number
Sex	
Male	85(61%)
Female	53(39%)
Age(year)	
20–40	13(9.4%)
40–60	29(21%)
60–80	65(47%)
> 80	31(22.6%)
BMI (kg/m2)	
< 18.5	14(10%)
18.5–24.9	63(45%)
25-29.9	54(39%)
30-34.9	7(6%)
Type of surgery	
intestinal anastomosis	87(63%)
enterostomy	51(37%)
SOFA score	
2–7	23(17%)
8–11	63(46%)
> 11	52(37%)
Pathogen type of culture	
Gram-positive	57(41%)
Gram-negative	29(21%)
none	52(38%)

### Comparisons of clinical data

There were no significant differences in white blood cell counts (WBC), procalcitonin (PCT), prothrombin time (PT), hematokrit (HCT), platelet lymphocyte ratio (PLR), neutrophil lymphocyte ratio (NLR) and C reactive protein (CRP) between the survival group and death group. The non-survivors were characterized as having lower platelet and fibrinogen. Meanwhile, the non-survivors were characterized as having higher creatinine, activated partial thromboplastin time (APTT), pro-BNP, D-dimer and liquid volume in first 24 h. The disease severity scores in non-survivor group were higher than survivor group. Moreover, the lactate levels in the non-survivors were significantly higher than in the survivors. The comparisons of these clinical datas are summarized in Table 2.

**Table 2:** Comparison of parameter among the survival and non-survival

Variable	Survivor group	Non-survivor group	P value
Age (y)	65.36 ± 16.91	70.56 ± 14.44	0.037
Sex (n)	94	44	0.558
Male	58	28	
Female	36	16	
Laboratory test			
Platelet (*10^9/L)	135.75 ± 71.89	85.75 ± 50.29	< 0.0001
White blood cell counts(*10^9/L)	10.45 ± 8.83	12.78 ± 10.55	0.860
Procalcitonin (ng/mL)	36.27 ± 36.81	40.80 ± 40.02	0.513
C reactive protein (mg/L)	235.76 ± 121.74	202.92 ± 144.60	0.354
Creatinine (µmol/L)	107.64 ± 64.02	185.27 ± 91.71	< 0.0001
Prothrombin time(s)	18.70 ± 5.10	20.74 ± 6.49	0.07
Activated partial thromboplastin time(s)	49.02 ± 12.40	71.86 ± 34.18	< 0.0001
Fibrinogen (g/L)	5.22 ± 2.62	3.07 ± 1.78	< 0.0001
D-dimer (µg/L)	5276.66 ± 4823.63	8622.77 ± 6121.30	0.001
Lactic acid (mmol/L)	3.55 ± 2.63	5.94 ± 4.34	0.001
Pro-BNP (pg/mL)	3960.81 ± 5050.60	11968.25 ± 5957.32	< 0.0001
Hematokrit (HCT)	0.31 ± 0.07	0.31 ± 0.59	0.985
Neutrophil lymphocyte ratio	30.13 ± 29.50	40.99 ± 44.82	0.096
Platelet lymphocyte ratio	434.96 ± 495.44	548.07 ± 587.97	0.414
Liquid volume (ml)			
In first 24 h	3759.86 ± 985.04	6366.36 ± 2949.31	0.001
In the third 24 h	3252.14 ± 837.92	3848.33 ± 2024.23	0.455
In the fifth 24 h	3411.56 ± 792.7	4144.17 ± 2159.30	0.531
Severity scores			
SOFA	9.39 ± 2.71	13.09 ± 3.08	< 0.0001
APACHEII	19.54 ± 6.20	27.61 ± 6.70	< 0.0001

SOFA: Sequential Organ Failure Assessment scores

APACHE II: Acute Physiology and Chronic Health Evaluation II

**Fig. 1:**
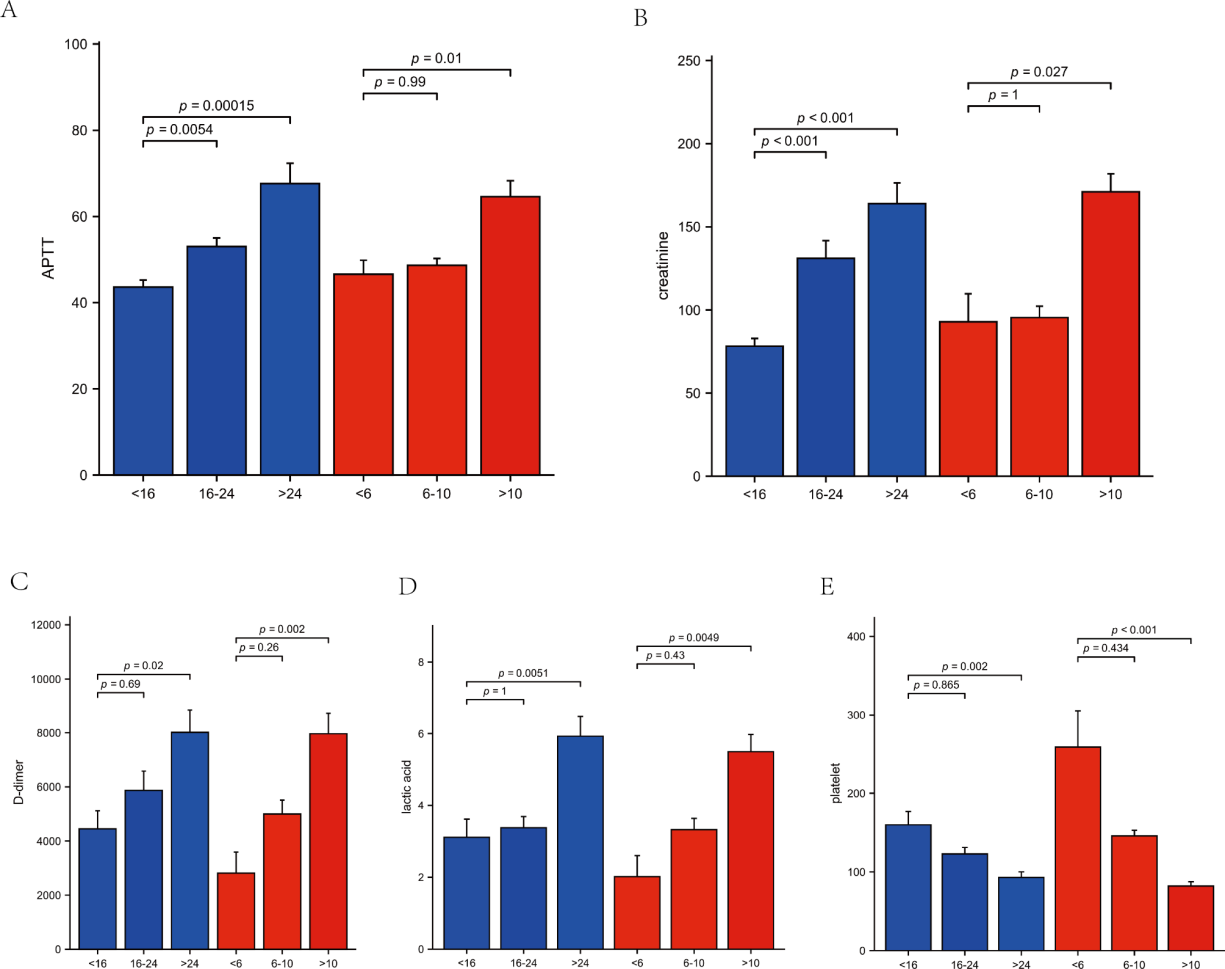
The serum levels of (**A**) APTT, (**B**) creatine, (**C**) D-dimer, (**D**)lactic acid and (**E**) platelet in septic shock patients caused by enteric perforation with different severity classifications. Red represents SOFA and blue represents APACHEII. APTT: Activated partial thromboplastin time. SOFA: Sequential Organ Failure Assessment scores APACHE II: Acute Physiology and Chronic Health Evaluation II

The histogram plots of the five clinical biomarkers graded by APACHE II and SOFA scores are displayed in Fig. 1. As SOFA scores and APACHEII scores incremented, these clinical indicators including APTT, D-dimer, lactate and creatinine increased and platelet decreased. The mortality of SOFA < 6, 6–10 and > 10 scores group were 0%, 16% and 52% respectively. The mortality of APACHEII < 16, 16–24 and > 24 scores group were 3%, 21.9% and 59.2% respectively.

### Significant predictors of the 28-day mortality in patients with septic shock caused by enteric perforation

In Table 3 of univariate Cox proportional hazards model, it was showed that APTT, PLT, Cr, D-dimer, Fibrinogen, Lac, age and Pro-BNP were significantly correlated with an increased probability of 28-day survival. In the Table 4 of multivariable Cox proportional hazards model, Lac, PLT, Fibrinogen, Cr and APTT were found to be associated with 28-day mortality. These factors were independent risk factors for septic shock caused by intestinal perforation.

**Table 3:** Univariate analysis

Variable	Hazard ratio	95% CI	P value
APTT	5.512	2.970-10.231	P < 0.0001
PLT	0.285	0.141–0.578	P < 0.0001
Cr	5.725	3.100-10.574	P < 0.0001
Ddimer	2.860	1.412–5.797	0.004
Fibrinogen	0.598	0.489–0.730	P < 0.0001
Lac	1.168	1.093–1.247	P < 0.0001
Age	1.743	0.912–3.331	0.093
Pro-BNP	2.933	1.622–5.304	P < 0.0001

APTT: Activated partial thromboplastin time

PLT: Platelet

Cr: Creatinine

Lac: Lactic acid

**Table 4:** Multivariate stepwise backward Cox regression analysis

Variable	Hazard ratio	95% CI	P value
Lac	2.087	1.056–4.125	0.034
PLT	0.453	0.215–0.954	0.037
Fibrinogen	0.580	0.455–0.741	P < 0.0001
Cr	3.292	1.705–6.356	P < 0.0001
APTT	3.550	1.806–6.979	P < 0.0001

APTT: Activated partial thromboplastin time

PLT: Platelet

Cr: Creatinine

Lac: Lactic acid

### The predictive efficacy of 28-day mortality

**Fig. 2:**
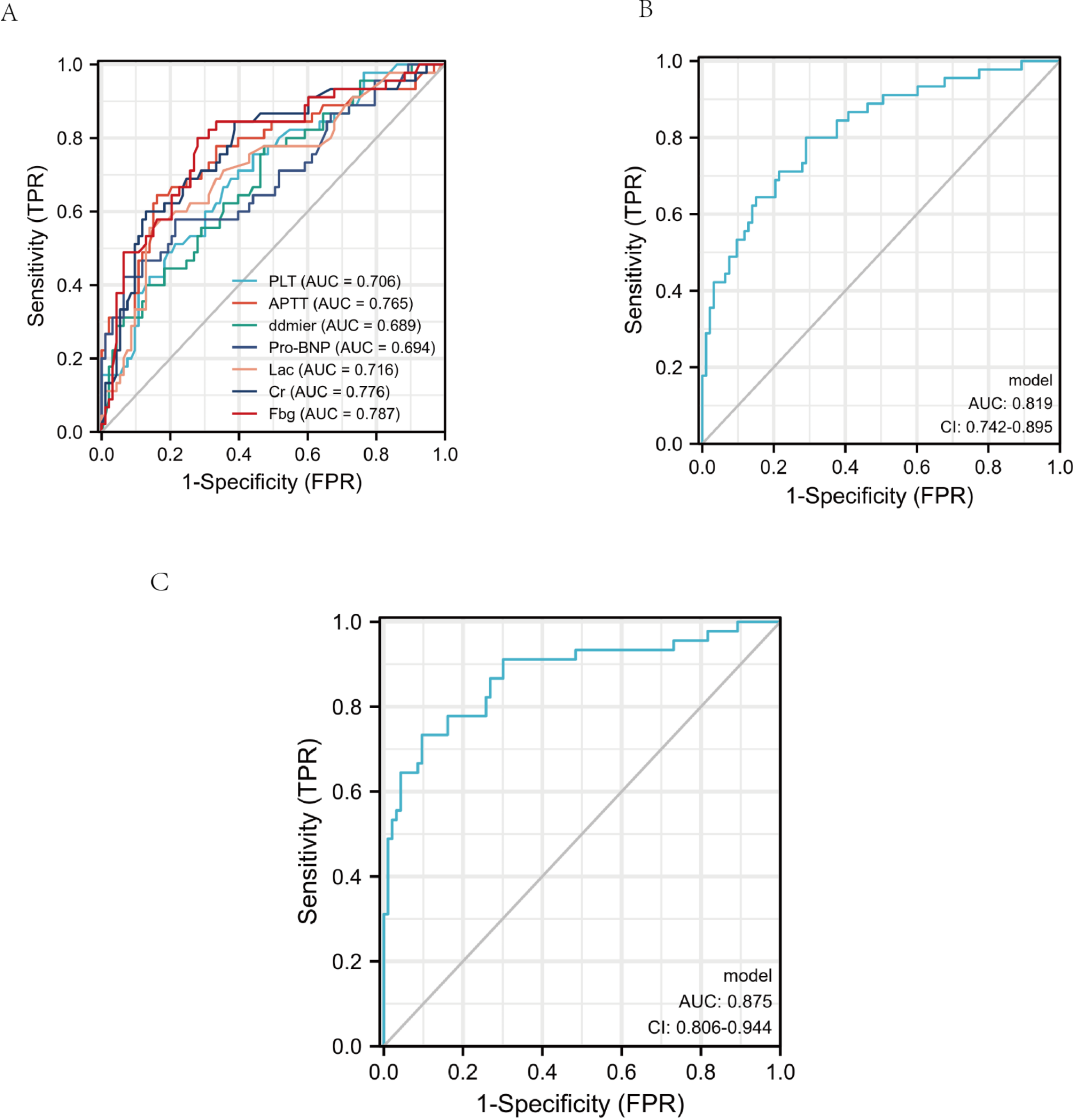
The ROC analysis of the studied biomarkers for predicting the development of 28-day mortality in septic shock patients caused by enteric perforation. (**A**) ROC analysis of fibrinogen, platelet, creatine, APTT, D-dimer, and lactic acid as a single parameter (**B**) ROC analysis of fibrinogen combined with APTT. (**C**) ROC analysis of fibrinogen plus creatine and APTT. APTT: Activated partial thromboplastin time

The ability of above clinical indicators to predict the prognosis of sepsis was evaluated using the receiver operating characteristic curve (ROC) analysis. The area under the curve (AUC) values of pro-BNP, D-dmier, Lac, APTT, Fibrinogen, PLT, Cr, Fibrinogen plus APTT and Fibrinogen plus APTT and Cr were 0.694 (0.594–0.793), 0.689 (0.595–0.784), 0.716 (0.621–0.811), 0.765 (0.675–0.856), 0.787 (0.702–0.862), 0.706 (0.614–0.798), 0.776 (0.689–0.862), 0.819 (0.742–0.895) and 0.875 (0.806–0.944) respectively. We compared AUC value among the different clinical indicators and found that Fibrinogen plus APTT and Cr presented the largest AUC value than the other variables. (Table 5; Fig. 2)

**Table 5:** The AUC of clinical biomarkers for predicting 28-day mortality

Variable	AUC	95% CI	Sensitivity	Specificity	Youden Index	Cut-off point
Pro-BNP	0.694	0.594–0.793	0.578	0.785	1.363	5225.50
ddimer	0.689	0.595–0.784	0.778	0.527	1.305	3730.00
Lac	0.716	0.621–0.811	0.556	0.860	1.416	4.85
APTT	0.765	0.675–0.856	0.839	0.659	1.483	55.55
Fibrinogen	0.787	0.702–0.862	0.800	0.720	1.520	3.965
PLT	0.706	0.614–0.798	0.756	0.559	1.315	120.50
Cr	0.776	0.689–0.862	0.600	0.871	1.471	184.50
Fbg /APTT	0.819	0.742–0.895	0.800	0.710	1.510	APTT = 44.8 Fbg = 3.59
Fbg /APTT/Cr	0.875	0.806–0.944	0.833	0.903	1.637	APTT = 51.6 Fbg = 1.99 Cr = 116

APTT: Activated partial thromboplastin time

PLT: Platelet

Cr: Creatinine

Lac: Lactic acid

**Fig. 3:**
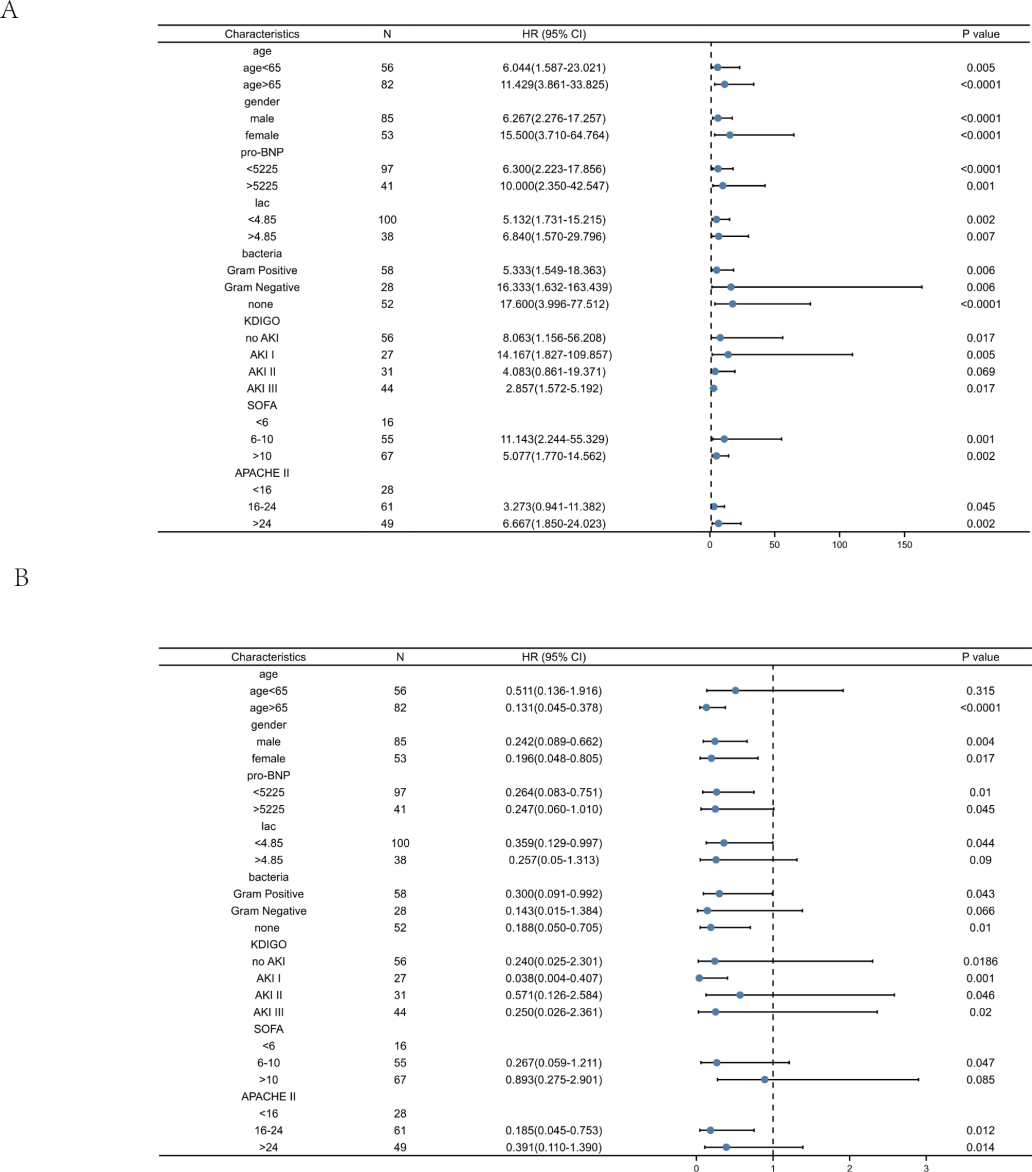
The forest plot of APTT and fibrinogen in a subgroup analysis. (**A**) The prognostic effect of APTT in each subgroup with different parameters (**B**) The prognostic effect of fibrinogen in each subgroup with different parameters. APTT: Activated partial thromboplastin time

The sensitivity, specificity, cut-off point and Youden index were calculated to assess the predictive value of each indicator comprehensively. We found that APTT had the highest sensitivity (0.839) and Fibrinogen plus APTT and Cr had the highest specificity (0.903) with a relatively high sensitivity (0.833) for 28-day mortality prediction. The cut-off point of each biomarker was listed in Table 5. Meanwhile, Fibrinogen plus APTT and Cr had the highest Youden index (1.637). In order to study the prognostic effect of APTT and fibrinogen in each subgroup with different parameters, we analyzed the prognostic effect of APTT and fibrinogen in each subgroup with different parameters. The subgroup analysis revealed that both APTT and fibrinogen were both key indicators of prognosis. (Fig. 3)

**Fig. 4:**
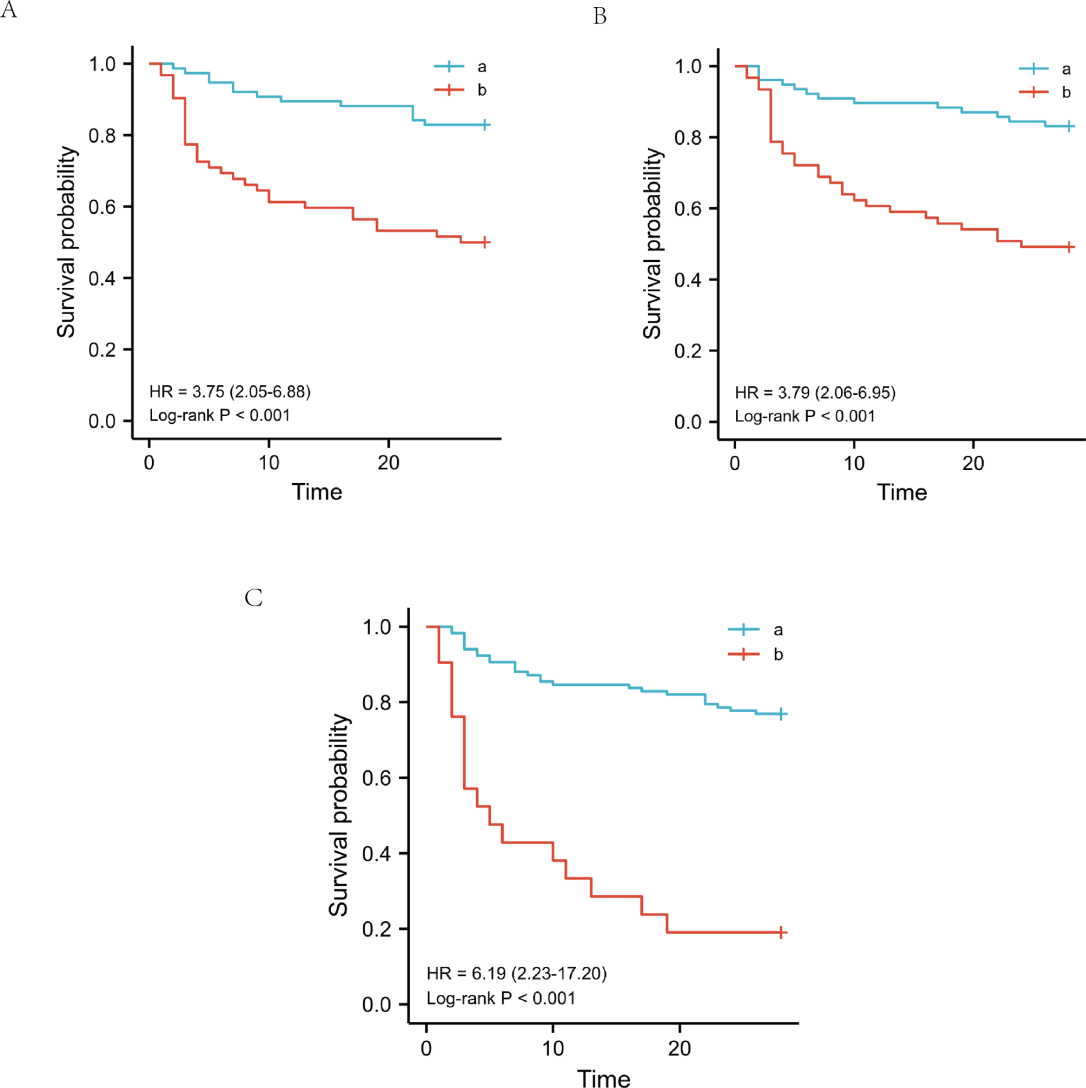
Kaplan–Meier plot showing survival in septic shock patients grouped by APTT, creatine, and fibrinogen levels. (**A**) Kaplan–Meier survival curves of 28-day mortality according to APTT levels. a = low APTT group (< 51.6), b = high APTT group (> 51.6). (**B**) Kaplan–Meier survival curves of 28-day mortality according to fibrinogen levels. a = high fibrinogen group (> 1.99), b = low fibrinogen group (< 1.99). (**C**) Kaplan–Meier survival curves of 28-day mortality according to creatine levels. a = low creatine group (< 116), b = high creatine group (> 116). APTT: Activated partial thromboplastin time. PLT: Platelet. Cr: Creatinine. Lac: Lactic acid

The 28-day mortality risk in septic shock patients caused by enteric perforation according to the cut-off point of fibrinogen, APTT and Cr were compared. Kaplan-Meier curve analysis indicated that the high APTT and Cr groups had a significantly higher 28-day mortality than the low APTT and Cr groups (Fig. 4A). Kaplan-Meier curve analysis also indicated that the low fibrinogen group had a significantly higher 28-day mortality than the high fibrinogen group (Fig. 4B). Kaplan-Meier curve analysis also indicated that the high Cr group had a significantly higher 28-day mortality than the low Cr group (Fig. 4C).

## Discussion

Septic shock is a known post-operative complication of enteric perforation, which can lead to multiple organ dysfunction and high mortality in the ICU [11]. As the primary site of inflammatory response, the endothelium not only activates the coagulation but also causes a decrease in platelets and the formation of microthrombus which further exacerbate vascular injury, leading to capillary leak [12]. Therefore, we noticed that more patients with septic shock caused by enteric perforation suffered from coagulation disorder and thrombocytopenia develop a higher SOFA score and APACHEII score, compared with patients with a less pronounced coagulation disorder. Using multivariable Cox regression analysis, we found that combination APTT, creatinine, and fibrinogen level on admission to the ICU were significant risk factors for the prognosis of septic shock. Therefore, the present study sought to use fibrinogen combined with creatinine and APTT as a death risk screening tool for septic shock caused by enteric perforation defined as, and the 28-day mortality predictive values of several biomarkers in septic shock caused by enteric perforation were evaluated and compared.

As is known by previous studies, platelets play an immunomodulatory role in the pathogenesis of septic shock in human patients [13]. When sepsis and septic shock occurs, the activated platelets form complexes with neutrophils and monocytes [14]. Moreover, activated platelets at the vascular endothelium not only produce chemokines but also provide an adhesive surface for both neutrophils and monocytes. Platelet-endothelial adhesion and platelet-leukocyte can lead to microthrombi in small vessels of microcirculatory. Immunothrombosis contributes to microvascular dysfunction, which is a hallmark of organ damage in sepsis [15]. The network of microvascular includes arterioles, venules and capillaries. In the development of septic shock, alteration of microvascular function caused by platelets correlates with the mortality and severity of septic shock [16]. Platelets are also associated with damage of multiple organs. Jason N Katz et al. showed that the patients with ARDS had excess numbers of platelets deposition in pulmonary vessels [17]. Platelets play an important role in acute kidney injury and septic cardiomyopathy [18, 19]. In this retrospective study, we demonstrated that platelets values were significantly lower in the non-survivors which was consistent with the previous studies [20–22]. But the predictive power of platelets is not useful for the 28-day mortality in sepsis.

Furthermore, direct exposure of the vascular endothelium to inflammatory factors and interaction with leukocytes mediates activation and injury of endothelial cells. Activated and damaged endothelial cells activate endogenous coagulation pathways, leading to enhanced procoagulant activity causing microthrombosis and capillary occlusion, further disrupting endothelial barrier integrity. When the integrity of the endothelial barrier is disrupted, tissue factor released from monocytes enters the bloodstream to participate in the activation of endogenous coagulation pathways, leading to coagulation dysfunction [23, 24]. This eventually leads to tissue ischemia and hypoxia causing organ dysfunction [25, 26]. Therefore, the biomarker (APTT) used to evaluate endogenous coagulation activity can effectively reflect the sepsis induced endothelial cell damage. In this retrospective study, we demonstrated that APTT was independently associated with high morbidity and mortality in patients with sepsis, which was consistent with the previous studies [27]. On the other hand, the fibrinogen level on admission was significantly lower in nonsurvivors than in survivors in our study. In general, fibrinogen is a marker indicative of depletion of hemostatic factors, reflecting an excessive hypercoagulable and hyperfibrinolytic state in sepsis-induced coagulopathy, and is considered an acute phase reactant, usually increased in patients with infection and inflammation [28]. High fibrinogen in the early stages of sepsis may reflect adaptation to infection and contribute to early recovery from sepsis. In contrast, low fibrinogen reflects a combination of depletion through microthrombosis and synthetic damage to the liver, which implies a worse outcome in septic shock. In this retrospective study, we demonstrated that fibrinogen was a valuable prognostic biomarker in patients with septic shock caused by enteric perforation, which was consistent with the previous studies [29]. Studies over the past decade have highlighted the central role of endothelial impairment-induced microcirculatory deficits in acute kidney injury in sepsis [30]. It is well known that the kidney is the organ with the richest vascular endothelium of all organs. High creatinine level in the early stages of sepsis may reflect the injury of vascular endothelium. In this retrospective study, we demonstrated that creatinine was a valuable prognostic biomarker in patients with septic shock caused by enteric perforation.

However, there was no significant difference in PCT and CRP levels between survivors and non-survivors. PCT (procalcitonin) is an acute-phase reactant that has been used to diagnose and potentially track the treatment of sepsis [31]. Some studies have shown that a higher level of PCT in patients often indicates a higher in-hospital mortality [32, 33]. However, in this study, the level of PCT was not significantly different between survivors and non-survivors. PCT did not elevate in viral infections and that serum levels of PCT would decrease following administration of appropriate antibiotic therapies [34]. From our COX analysis, we observed that PCT was not a better index to evaluate the prognosis of sepsis patients. In a of meta-analysis [35], concluded that the PCT value on day 1 was not found to be significantly different between patients with severe sepsis and septic shock. However, the issue of PCT still needs to be paid more attention, and there may be some bias in our study leading to this result. In a observational and prospective study conducted by Mierzchala-Pasierb M et al. also showed that there was no significant difference for the CRP levels between any subgroups according to disease seriousness [36]. In addition, CRP was lack of specificity to diagnose bacterial versus non-bacterial infections accurately [34]. Therefore, CRP is not a sufficient parameter for prognostic evaluation. Blood fibrinogen, creatinine, and APTT are all clinically easily accessible biomarkers which are closely related to the occurrence and development of sepsis. There may be of great value for the treatment of septic shock to explore the deep meaning and hidden important clinical information of fibrinogen plus APTT and creatinine.

There are several important limitations in this study. First, this was a retrospective analysis in a single center. A prospective multicenter study to conform this conclusion is needed in future. Second, the size of the study population was small because we had a strict inclusion and exclusion criteria to decrease the bias among patients. With this method, the composition of patients was more homogeneous and we can provide more specific information to clinicians about septic shock caused by enteric perforation. Therefore, further larger prospective studies and intervention trials is needed to identity the risk factors of septic shock caused by enteric perforation. Despite there are several limitations in this study, we believe that our findings are important for the clinicians to early identify patients with life-threatening septic shock caused by enteric perforation.

## Conclusions

The present study provides new insights into patients with septic shock caused by enteric perforation. By analyzing the patient data, our study indicated that the levels of fibrinogen, APTT, and Cr on admission to the ICU were associated with the severity of septic shock caused by enteric perforation. These indicators can be easily obtained, most clinicians can make good use of, especially in developing countries where most cases of gastrointestinal perforation are.

### Electronic supplementary material

Below is the link to the electronic supplementary material.

Supplementary Material 1

## Data Availability

All data generated and analysed during this study are included in this published article and its supplementary information files.

## Declarations

### Competing of interests

All the authors do not have any Competing of Interest to declare.
